# The association between lower prognostic nutritional index and higher short- & long-term mortality in older adults (≥ 70 years) undergoing coronary artery bypass grafting: a retrospective study

**DOI:** 10.1186/s12877-025-05833-9

**Published:** 2025-03-14

**Authors:** Zihua Liu, Zikun Wang, Qi Huang, Bo Hu, Mingliang Li, Yilin Pan, Yangyang Sun, Hao Cao, Kai Xu, Lei Yang, Zhi Li, Yangyang Zhang, Xin Zhao

**Affiliations:** 1 Department of Cardiovascular Surgery, Qilu Hospital of Shandong University, Jinan, P. R. China; 2 Department of Cardiovascular Surgery, Shanghai Chest Hospital, Shanghai Jiao Tong University School of Medicine, Shanghai, P. R. China; 3 Department of Anesthesiology, Shanghai Chest Hospital, Shanghai Jiao Tong University School of Medicine, Shanghai, P. R. China; 4 Department of Cardiology, Shanghai East Hospital, School of Medicine, Tongji University, Shanghai, P. R. China; 5 Department of Cardiovascular Surgery, the General Hospital of Ningxia Medical University, Yinchuan, P. R. China; 6 The First School of Clinical Medicine, Nanjing Medical University, Nanjing, P. R. China; 7 Department of Cardiovascular Surgery, Shanghai East Hospital, School of Medicine, Tongji University, Shanghai, P. R. China; 8 Department of Cardiovascular Surgery, The First Affiliated Hospital of Nanjing Medical University, Nanjing, P. R. China

**Keywords:** Prognostic nutritional index, Older adults, Mortality, Coronary artery bypass grafting

## Abstract

**Background:**

The incidence of cardiovascular diseases among old individuals is on the rise with the growing trend of population aging. Coronary artery bypass grafting (CABG) is an important treatment modality for coronary heart diseases and is increasingly employed in older adults. However, concerns arise due to the poor prognosis following surgery in this population. The prognostic nutritional index (PNI) reflects the nutritional status and immune function of patients. It has been previously utilized in prognostic assessments for other surgical procedures and receives increasing attention in the field of cardiovascular surgery.

**Methods:**

This retrospective study examined a cohort of older adults (70 to 90 years) who underwent initial CABG-only surgery at five cardiac centers, excluding patients with coexisting neoplastic or immune disorders. The objective was to investigate the relationship between low PNI and both short- and long-term mortality in this population. PNI was calculated based on total lymphocyte count and serum albumin concentration measured before surgery, after surgery, and before discharge. The cut-off value of PNI was established through receiver’s operating characteristic curve. Univariate and multivariate logistic and cox regression analyses were performed to identify the independent risk factors related to the occurrence of short- and long-term mortality. Smooth survival model and Kaplan-Meier analysis were employed to evaluate survival and relative risk.

**Results:**

Among the 1173 patients, 90 patients (7.7%) experienced short-term mortality and 131 (11.2%) patients had long-term mortality during follow-up and the survival probabilities at 1,3,5,10 years were 96.98%, 94.64%, 89.89%, 76.96%, respectively. In this population, lower preoperative PNI was independently and significantly correlated with short-term mortality (OR = 2.372, 95%CI: 1.394–4.035). Additionally, a low PNI before discharge was independently and significantly associated with increased long-term mortality risk in older adults who underwent CABG (HR = 1.451, 95%CI: 1.012–2.082). Long-term follow-up also showed that patients with a low PNI before discharge had significantly higher long-term mortality (log-rank: *P* = 0.004). Moreover, extended Kaplan-Meier analysis showed that women (log-rank: *P* = 0.005) and obese patients (log-rank: *P* = 0.073) appeared to have higher long-term survival rates.

**Conclusion:**

The current investigation unveiled that PNI has emerged as an autonomous determinant for both short-and long-term mortality in older adults receiving CABG.

## Background

In recent decades, the prevalence of cardiovascular and cerebrovascular diseases has persisted at high levels both in China and worldwide, coinciding with socioeconomic development and population aging [1, 2]. Coronary artery bypass grafting (CABG) is a crucial surgical intervention employed to address serious coronary heart disease (CHD) [3, 4]. With the improvement in living and healthcare standards, the average life expectancy in China has increased to 77 years old. Consequently, the number of older adults undergoing CABG is rising year by year. Despite improvements in surgical techniques and perioperative cure, older adults experience a compounded effect of CHD and surgical trauma, leading to elevated rates of postoperative complications and surgical mortality [5].

An increasing number of studies are no longer confined to searching for independent risk factors of mortality after CABG with postoperative complications [6–8]. Instead, researchers are shifting to specific physical indicators. The prognostic nutritional index (PNI) [9] serves as a measure for chronic inflammation, immune system functionality, and nutritional well-being. Its computation relies on the serum albumin concentration and total lymphocyte count in peripheral blood, offering potential for prognosticating patient outcomes. A previous study has characterized PNI as a direct and unbiased indicator of unfavorable outcomes [10]. Nevertheless, in older adults, the association between low PNI and mortality rates in the short- and long-term following CABG remains a topic of debate.

The aim of this study was to investigate the relationship between perioperative PNI and short- and long-term mortality after CABG in the older adults.

## Materials and methods

### Patients

**Fig. 1:**
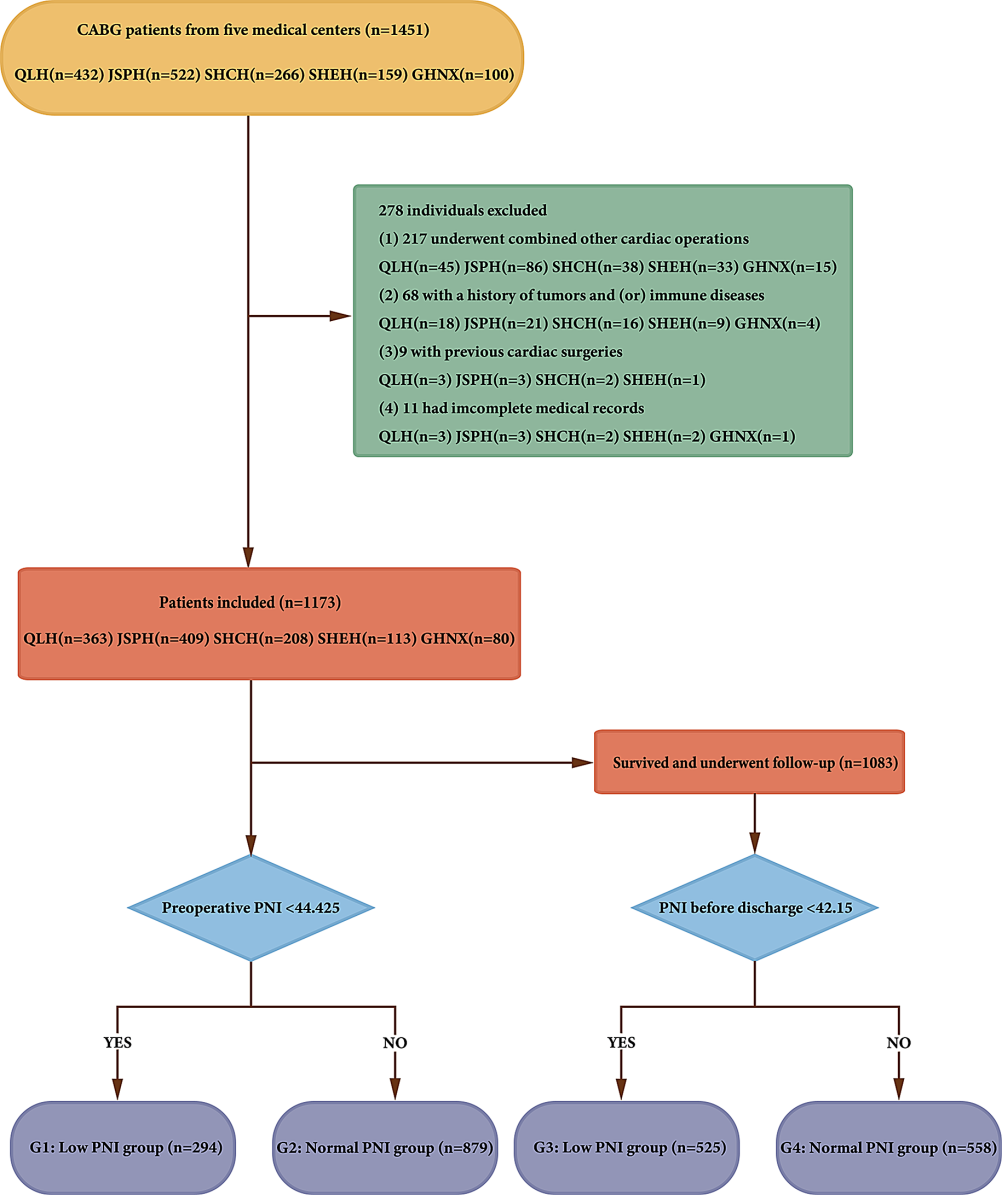
Flow chart of the study. CABG, coronary artery bypass grafting; QLH, Qilu Hospital of Shandong University; JSPH, Jiangsu Provincial Hospital; SHCH, Shanghai Chest Hospital; SHEH, Shanghai East Hospital; GHNX, General Hospital of Ningxia Medical University; PNI, prognostic nutritional index

This retrospective analysis comprised a consecutive series of 1451 patients aged 70 to 90 years with isolated CABG from five medical centers (Qilu Hospital of Shandong University - QLH, Jiangsu Provincial Hospital - JSPH, Shanghai Chest Hospital - SHCH, Shanghai East Hospital - SHEH, General Hospital of Ningxia Medical University - GHNX). Patients were excluded if they met any of the following criteria: (1) undergoing other cardiac surgeries; (2) history of tumors and (or) immune diseases; (3) incomplete medical records; (4) previous cardiac surgeries. After a thorough process of inclusion and exclusion, the initial database consisted of 1173 patients (363 from QLH, 409 from JSPH, 208 from SHCH, 113 from SHEH, 80 from GHNX) for the purpose of this study. The inclusion and exclusion flowcharts were shown in Fig. 1.

Venous blood samples were collected from all patients to perform routine liver function and blood count measurements. These samples, approximately 2–4 ml each, were obtained 48 h before operation, 24 h after operation, and before discharge, following standardized procedures carried out by nursing staff. The central laboratories of the five hospitals measured albumin concentrations and total peripheral blood lymphocyte counts according to unified standards. The perioperative demographic data were also recorded. The enrolled patients were categorized by the predetermined cut-off value into a low PNI group and a normal PNI group.

### Definition of key indicators

#### PNI

PNI was calculated as follows: 10* serum albumin (g/dl) + 0.005 * total lymphocyte count (mm^3^) [11].

#### Short-term mortality

Short-term mortality criteria employed here were specifically defined as in-hospital mortality encompassing deaths occurring during or within 30 days following surgical procedures.

#### Long-term mortality

Long-term mortality was defined as mortality events observed during extended post-discharge monitoring, encompassing cardiac etiologies, neoplasms, and infectious diseases, among other factors.

#### Sex and obesity status

Sex was categorized as male or female, and obesity was classified based on BMI values into lean (< 18.5 kg/m²), normal weight (18.5–23.9 kg/m²), overweight (23.9–27.9 kg/m²), obesity (27.9–30 kg/m²), and severe obesity (> 30 kg/m²). Overweight, obesity and severe obesity were defined as obesity.

### Outcomes

The primary outcome was the relationship between low preoperative PNI and short-term mortality, while the secondary outcome focused specifically on the impact of low PNI before discharge on long-term mortality. In addition, we defined exploratory outcomes to investigate potential variations in survival based on sex and obesity status.

### Informed consent

In this retrospective study, measures were implemented to safeguard patient confidentiality and uphold ethical principles. With the endorsement and consent of the corresponding ethics committees, all enrolled patients were granted exemption from the necessity of informed consent for their involvement in the study. The execution of this study adhered to the ethical norms and guidelines established by the five hospital ethics committees, with registration numbers KYLL202306033, 2023SR034, IS23006, 2023134 and KYLL20230004, respectively.

### Statistical analysis

Continuous variables were presented as mean ± standard deviation and analyzed using Student’s t-tests, while non-normally distributed variables were represented by medians and interquartile range (IQR). Categorical variables were reported as numbers and percentages, and analyzed via Chi-square or Fisher’s exact tests. Receiver’s operating characteristic (ROC) curves and Youden index were used to determine the optimal cut-off point for detecting low and normal preoperative PNI. Short-term mortality risk factors were identified using both univariate and multivariate logistic regression analyses. Long-term mortality risk factors were identified using univariate and multivariate cox regression analyses. Significant variables in the univariate analysis, along with clinically recognized risk factors, were included in the multivariate analysis to avoid overfitting. Variables with *P* < 0.1 in the univariate analysis were included in the multivariate logistic model. Variables with *P* < 0.05 in the univariate analysis were included in the multivariate Cox model. Odds ratios (ORs) with corresponding 95% confidence intervals (CIs) were reported for these analyses. Survival analysis was conducted using Kaplan-Meier survival curves, and the log-rank test was done to compare survival between the low and normal PNI groups before discharge, the male and female groups, and the obesity and non-obesity groups.

In all statistical analyses, the predetermined significance level was *P* < 0.05 using a two-sided approach. GraphPad Prism 9.0 (GraphPad Software, CA) and R 4.3.0 (http://www.R-project.org) with the R package “rstpm2” were utilized to generate figures, and SPSS 25.0 was used for data analysis.

## Results

### Baseline clinical characteristics

In the total cohort, the mean age was 73.32 years and the range of follow-up time was 1 to 225.8 months. Among the patients, 848 (72.3%) were men, 793 (67.6%) had hypertension, 407 (34.7%) had diabetes, 29 (2.5%) underwent emergency surgery, 77 (6.6%) had history of percutaneous coronary intervention (PCI), 90 (7.7%) experienced short-term mortality, and 131 (11.2%) died during long-term follow-up. The baseline clinical characteristics are presented in Table 1.

**Table 1:** Baseline patient characteristics, clinical and disease characteristics according to G1 and G2

	Total	G1 (PNI < 44.425)	G2 (PNI ≥ 44.425)
	*N* = 1173	*n* = 294	*n* = 879
Age (year)	73.32 ± 3.32	73.49 ± 3.43	73.27 ± 3.29
Male (n, %)	848(72.3)	236(80.3)	612(69.6)
Weight (kg)	66.93 ± 9.49	65.69 ± 9.12	67.35 ± 9.59
Hight (cm)	166.07 ± 7.48	166.46 ± 7.18	165.93 ± 7.58
Creatinine (umol/L)	82.62 ± 30.98	90.25 ± 44.31	80.07 ± 24.50
Ccr (mL/min)	68.49 ± 19.88	64.40 ± 20.75	69.86 ± 19.41
Preoperative LVEF (%)	58.45 ± 9.80	56.78 ± 11.08	59.01 ± 9.27
Number of diseased coronary artery	2.84 ± 0.46	2.82 ± 0.49	2.85 ± 0.45
BMI (kg/m^2^)	24.27 ± 3.00	23.68 ± 2.82	24.46 ± 3.04
BSA (m^2^)	1.83 ± 0.15	1.82 ± 0.14	1.84 ± 0.15
Number of graft vessel	3.19 ± 0.90	3.15 ± 0.89	3.20 ± 0.90
Euro	2.67 ± 3.63	3.64 ± 5.43	2.34 ± 2.72
Preoperative serum albumin (g/L)	39.70 ± 4.17	35.05 ± 3.22	41.25 ± 3.18
Preoperative TLC (×10^9^/L)	1.63 ± 0.59	1.19 ± 0.39	1.78 ± 0.58
Postoperative serum albumin (g/L)	33.72 ± 5.83	31.42 ± 5.94	34.92 ± 6.00
Postoperative TLC (×10^9^/L)	0.91 ± 0.60	0.79 ± 0.49	0.95 ± 0.63
Serum albumin before discharge (g/L)	35.42 ± 4.38	34.50 ± 4.75	35.72 ± 4.21
TLC before discharge (×10^9^/L)	1.35 ± 0.58	1.23 ± 0.60	1.39 ± 0.57
Preoperative PNI	47.85 ± 5.47	41.02 ± 3.22	50.13 ± 3.96
Postoperative PNI	38.26 ± 6.76	35.39 ± 6.33	39.22 ± 6.62
PNI before discharge	42.17 ± 5.27	40.63 ± 5.44	42.69 ± 5.11
Long-term survival time (months)	61.79 ± 39.88	57.53 ± 39.94	63.09 ± 39.80
NYHA classification (n, %)			
I	189(16.1)	36(12.2)	153(17.4)
II	520(44.3)	120(40.8)	400(45.5)
III	414(35.3)	114 (38.8)	300(34.1)
IV	50(4.3)	24(8.3)	26(3.0)
Obesity status (n, %)			
Lean	17(1.4)	6(2.0)	11(1.3)
Normal weight	540(46.0)	150(51.0)	390(44.4)
Overweight	504(43.0)	120(40.8)	384(43.7)
Obesity	71(6.1)	11(3.7)	60(6.8)
Severe obesity	41(3.5)	7(2.4)	34(3.9)
CHD classification (n, %)			
Stable	284(24.2)	73(24.8)	211(24.0)
Unstable	767(65.4)	167(56.8)	600(68.3)
Myocardial infarction	122(10.4)	54(18.4)	68(7.7)
Hypertension (n, %)	793(67.6)	179(60.9)	614(69.9)
Diabetes (n, %)	407(34.7)	102(34.7)	305(34.7)
Cerebrovascular disease (n, %)			
Lacunar infarction	132(11.3)	27(9.2)	105 (11.9)
Cerebral infarction	98(8.4)	28(9.5)	70(8.0)
PVD (n, %)	119(10.1)	44(15.0)	75(8.5)
Emergency surgery (n, %)			
Emergency	29(2.5)	11(3.7)	18(2.0)
Rescue	9(0.8)	6(2.0)	3(0.3)
Valvular diseases (n, %)	146(12.4)	44(15.0)	102(11.6)
IABP implantation (n, %)			
Preoperative	27(2.3)	21(7.1)	6(0.7)
Intraoperative	28(2.4)	10(3.4)	18(2.0)
Postoperative	17(1.4)	4(1.4)	13(1.5)
COPD (n, %)	56(4.8)	21(7.1)	35(4.0)
Preoperative AF (n, %)	42(3.6)	9(3.1)	33(3.8)
Pulmonary hypertension (n, %)	326(27.8)	82(27.9)	244(27.8)
History of PCI (n, %)	77(6.6)	21(7.1)	56(6.4)
Renal failure (n, %)	31(2.6)	15(5.1)	16(1.8)
Postoperative short-term mortality (n, %)	90(7.7)	41(13.9)	49(5.6)
CPB (n, %)	205(17.5)	53(18.0)	152(17.3)

Ccr, endogenous creatinine clearance rate; LVEF, left ventricular ejection fraction; BMI, body mass index; BSA, body surface area; TLC, total peripheral blood lymphocyte count; PNI, prognostic nutritional index; NYHA, New York heart association; CHD, coronary heart disease; PVD, peripheral vascular disease; IABP, Intra-aortic balloon pump; COPD, chronic obstructive pulmonary disease; AF, atrial fibrillation; PCI, percutaneous coronary intervention; CPB, cardiopulmonary bypass

Values are mean ± SD or n (%)

### Groups of PNI

To explore the association between perioperative PNI alterations and short- or long-term prognosis in patients undergoing CABG, this study recommended categorizing patients into groups based on PNI at various time points. With the ROC curve and the Youden index, 44.425 was identified as the optimal cut-off value. Preoperative PNI values below 44.425 were classified as low preoperative PNI group (G1, *n* = 294), while values equal to or above 44.425 were classified as normal preoperative PNI group (G2, *n* = 879). Demographic, clinical, and laboratory data for G1 and G2 are outlined in Table 1. The group with lower preoperative PNI, G1, exhibited reduced weight, creatinine clearance rate (Ccr), preoperative left ventricular ejection fraction (LVEF), and body mass index (BMI), as well as elevated creatinine and euro score levels. Conversely, G1 displayed a higher prevalence of myocardial infarction, peripheral vascular disease (PVD), emergency surgery, intra-aortic balloon pump (IABP) implantation, chronic obstructive pulmonary disease (COPD), renal failure, and postoperative short-term mortality.

Patients who survived surgery and underwent follow-up were divided into a low PNI before discharge group (G3, *n* = 525) or a normal PNI before discharge group (G4, *n* = 558) based on the median PNI before discharge (42.15). Demographic, clinical, and laboratory data for G3 and G4 groups are presented in Table 2. G3 demonstrated lower Ccr and BMI values, were older and taller, had higher levels of creatinine and euro scores, and received more graft vessels. Additionally, G3 had lower rates of female representation and obesity.

**Table 2:** Baseline patient characteristics, clinical and disease characteristics according to G3 and G4

	Total	G3 (PNI < 42.15)	G4 (PNI ≥ 42.15)
	*N* = 1083	*n* = 525	*n* = 558
Age (year)	73.30 ± 3.27	73.59 ± 3.45	73.00 ± 3.06
Male (n, %)	793(73.2)	424(78.4)	369(68.1)
Weight (kg)	67.20 ± 9.41	67.35 ± 9.15	67.05 ± 9.67
Hight (cm)	166.25 ± 7.39	167.29 ± 6.80	165.61 ± 7.66
Creatinine (umol/L)	81.00 ± 26.76	83.98 ± 31.91	78.03 ± 19.95
Ccr (mL/min)	69.38 ± 19.38	68.18 ± 19.53	70.58 ± 19.16
Preoperative LVEF (%)	58.85 ± 9.46	59.54 ± 8.98	58.16 ± 9.88
Number of diseased coronary artery	2.85 ± 0.45	2.86 ± 0.45	2.85 ± 0.45
BMI (kg/m^2^)	24.31 ± 2.98	24.13 ± 2.84	24.49 ± 3.11
BSA (m^2^)	1.84 ± 0.15	1.84 ± 0.14	1.83 ± 0.15
Number of graft vessel	3.22 ± 0.89	3.32 ± 0.95	3.12 ± 0.84
Euro	2.33 ± 2.20	2.42 ± 2.30	2.25 ± 2.10
Preoperative serum albumin(g/L)	39.82 ± 4.13	39.20 ± 4.22	40.44 ± 3.94
Preoperative TLC (×10^9^/L)	1.64 ± 0.58	1.56 ± 0.58	1.72 ± 0.57
Postoperative serum albumin(g/L)	33.93 ± 5.98	33.23 ± 5.87	34.64 ± 6.01
Postoperative TLC (×10^9^/L)	0.91 ± 0.60	0.89 ± 0.58	0.93 ± 0.63
Serum albumin before discharge(g/L)	35.67 ± 4.11	32.91 ± 2.75	38.41 ± 3.32
TLC before discharge (×10^9^/L)	1.37 ± 0.55	1.14 ± 0.40	1.60 ± 0.58
Preoperative PNI	48.02 ± 5.35	47.02 ± 5.31	49.02 ± 5.20
Postoperative PNI	38.49 ± 6.58	37.70 ± 6.58	39.29 ± 6.49
PNI before discharge	42.51 ± 4.94	38.60 ± 2.76	46.42 ± 3.25
Long-term survival time	61.79 ± 39.88	61.77 ± 39.78	63.11 ± 41.78
NYHA classification (n, %)			
I	186(17.2)	89(16.5)	97(17.9)
II	489(45.2)	268(49.5)	221(40.8)
III	374(34.5)	174(32.2)	200(36.9)
IV	34(3.1)	10(1.8)	24(4.4)
Obesity status (n, %)			
Lean	14(1.3)	11(2.0)	3(0.6)
Normal weight	498(46.0)	255(47.1)	243(44.8)
Overweight	468(43.1)	230(42.5)	237(43.7)
Obesity	67(6.2)	34(6.3)	33(6.1)
Severe obesity	37(3.4)	11(2.0)	26(4.8)
CHD classification (n, %)			
Stable	263(24.3)	158(30.6)	105(19.4)
Unstable	716(66.1)	323(59.7)	393(72.5)
Myocardial infarction	104(9.6)	60(11.1)	44(8.1)
Hypertension (n, %)	731(67.5)	378(69.9)	353(65.1)
Diabetes (n, %)	366(33.8)	171(31.6)	195(36.0)
Cerebrovascular disease (n, %)			
Lacunar infarction	125(11.5)	72(13.3)	53(9.8)
Cerebral infarction	84(7.8)	30(5.5)	54(10.0)
PVD (n, %)	109(10.1)	56(10.4)	53(9.8)
Emergency surgery (n, %)			
Emergency	22(2.0)	11(2.0)	11(2.0)
Rescue	2(0.2)	1(0.2)	1(0.2)
Valvular diseases (n, %)	133(12.3)	74(13.7)	59(10.9)
IABP implantation (n, %)			
Preoperative	14(1.3)	8(1.5)	6(1.1)
Intraoperative	17(1.6)	8(1.5)	9(1.7)
Postoperative	4(0.4)	3(0.6)	1(0.2)
COPD (n, %)	46(4.2)	27(5.0)	19(3.5)
Preoperative AF (n, %)	40(3.7)	19(3.5)	21(3.9)
Pulmonary hypertension (n, %)	299(27.6)	133(25.3)	162(29.9)
History of PCI (n, %)	64(5.9)	36(6.7)	28(5.2)
Renal failure (n, %)	18(1.7)	11(2.0)	7(1.3)
CPB (n, %)	173(16.0)	79(14.6)	94(17.3)

Ccr, endogenous creatinine clearance rate; LVEF, left ventricular ejection fraction; BMI, body mass index; BSA, body surface area; TLC, total peripheral blood lymphocyte count; PNI, prognostic nutritional index; NYHA, New York heart association; CHD, coronary heart disease; PVD, peripheral vascular disease; IABP, Intra-aortic balloon pump; COPD, chronic obstructive pulmonary disease; AF, atrial fibrillation; PCI, percutaneous coronary intervention; CPB, cardiopulmonary bypass

Values are mean ± SD or n (%)

### Impact of risk factors on short-term mortality

**Fig. 2:**
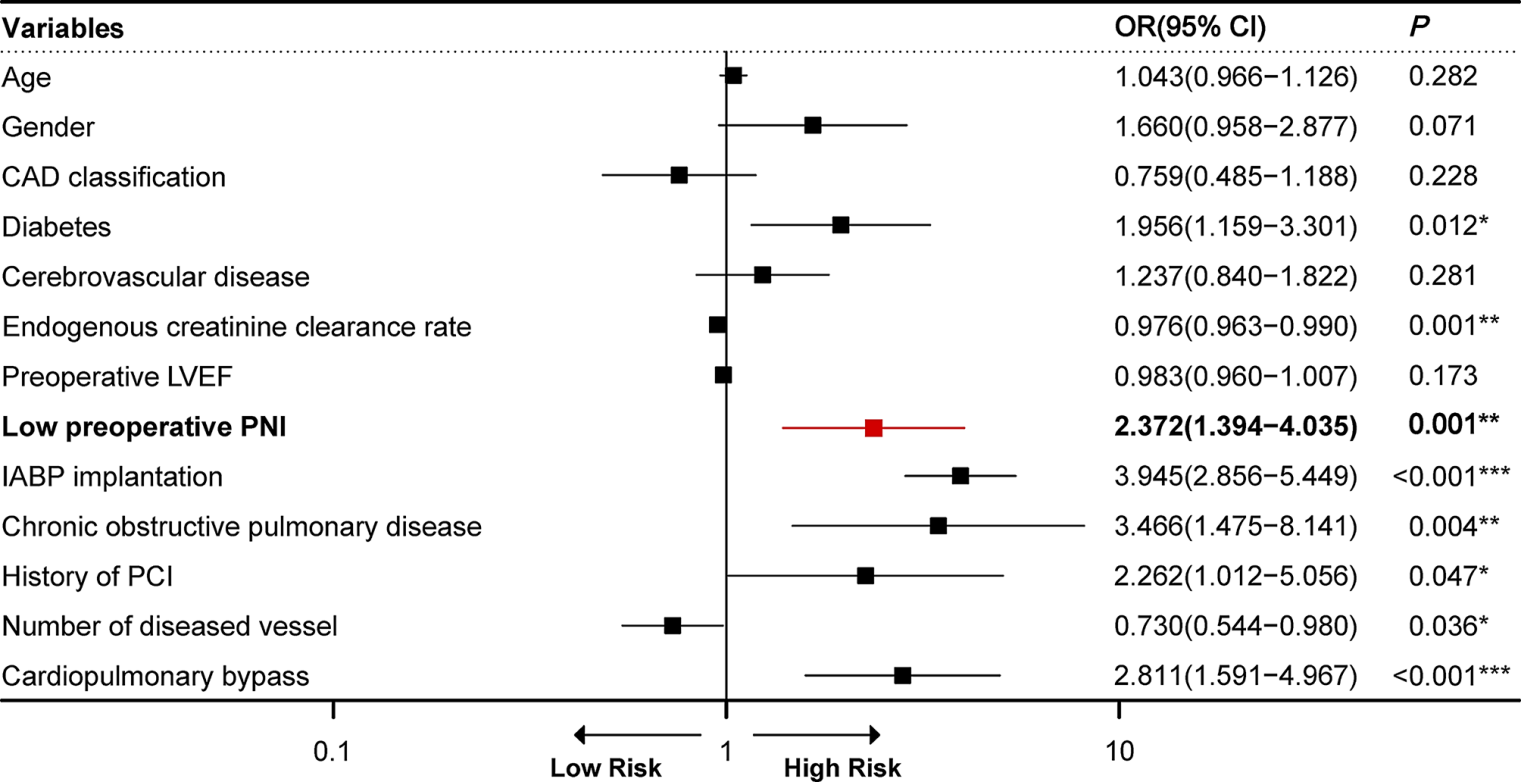
Forest plot of multivariate logistic regression model for short-term mortality of older adults with CABG. The variables are represented with log-odds along with their confidence intervals. CAD, coronary artery disease; LVEF, left ventricular ejection fractions; PNI, prognostic nutritional index; IABP, intra-aortic balloon pump; PCI, percutaneous coronary intervention; OR, odds ratio; CI, confidence interval. *P < *0.05*, **P < *0.01*, ***P < *0.001*

Univariate logistic analysis was conducted to identify the factors that were significantly associated with an increased risk of short-term mortality. The significant factors included preoperative PNI, gender, diabetes, Ccr, COPD, preoperative LVEF, IABP implantation, history of PCI, number of diseased vessels, and cardiopulmonary bypass (CPB) (Table 3). Additional multivariate logistic regression analysis revealed that low preoperative PNI (OR = 2.372, 95%CI: 1.394–4.035, *P* = 0.001), diabetes, Ccr, IABP implantation, COPD, history of PCI, number of diseased vessels, and CPB were independent risk factors of short-term mortality (Fig. 2).

**Table 3:** Univariate logistic regression analysis for short-term mortality

Variables	OR	95%CI for OR Lower-Upper	*P* value
Age	1.029	0.967–1.095	0.373
Gender	1.740	1.116–2.714	0.015
Ccr	0.968	0.956–0.979	< 0.001
Preoperative LVEF	0.956	0.939–0.974	< 0.001
Number of diseased coronary artery	0.536	0.373–0.770	0.001
BMI	0.931	0.862–1.005	0.068
Number of graft vessel	0.579	0.453–0.741	< 0.001
Preoperative PNI	0.928	0.893–0.965	< 0.001
Postoperative PNI	0.931	0.900-0.964	< 0.001
NYHA classification	2.518	1.863–3.402	< 0.001
Obesity status	0.978	0.816–1.173	0.812
CHD classification	1.416	0.971–2.066	0.071
Hypertension	1.066	0.670–1.696	0.786
Diabetes	1.639	1.063–2.529	0.025
Cerebrovascular disease	1.323	0.969–1.805	0.078
PVD	1.117	0.562–2.219	0.752
Emergency surgery	5.743	3.279–10.025	< 0.001
Valvular diseases	1.206	0.652–2.231	0.551
IABP implantation	4.234	3.171–5.652	< 0.001
COPD	2.818	1.371–5.793	0.005
Preoperative AF	0.593	0.141–2.493	0.475
Pulmonary hypertension	1.124	0.702–1.798	0.627
History of PCI	2.688	1.418–5.096	0.002
CPB	2.902	1.830–4.603	< 0.001

OR, odds Ratio; CI, confidence interval; Ccr, endogenous creatinine clearance rate; LVEF, left ventricular ejection fractions; BMI, body mass index; PNI, prognostic nutritional index; NYHA, New York heart association; CHD, coronary heart disease; PVD, peripheral vascular disease; IABP, intra-aortic balloon pump; COPD, chronic obstructive pulmonary disease; AF, atrial fibrillation; PCI, percutaneous coronary intervention; CPB: cardiopulmonary bypass

### Impact of risk factors on long-term mortality

**Fig. 3:**
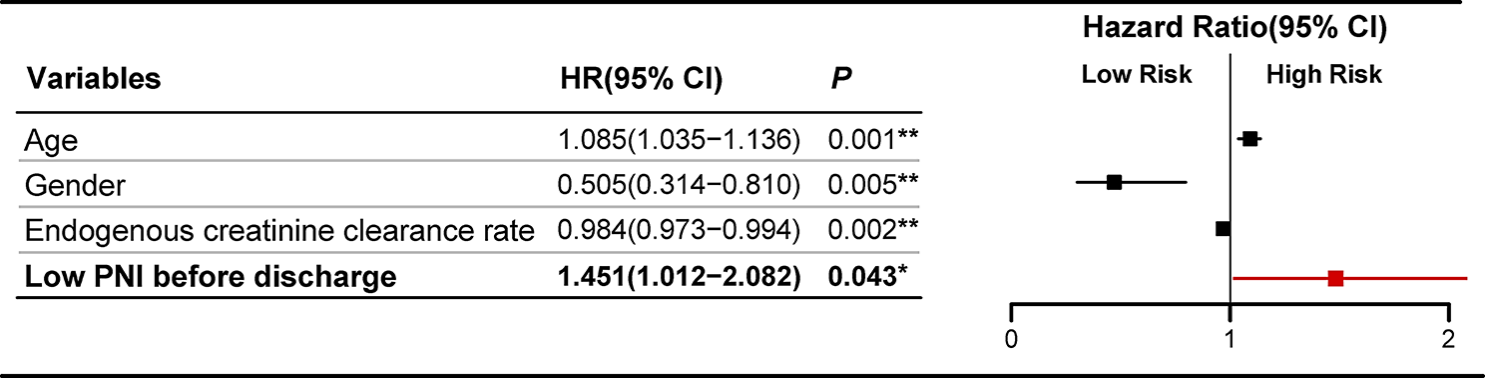
Forest plot of hazard ratios (HR) for variables associated with long-term mortality in older adults after CABG. HR and 95% confidence intervals (CI) were obtained by Multivariate Cox regression analysis. PNI, prognostic nutritional index; HR, hazard ratio; CI, confidence interval. *P < *0.05*, **P < *0.01*

**Fig. 4:**
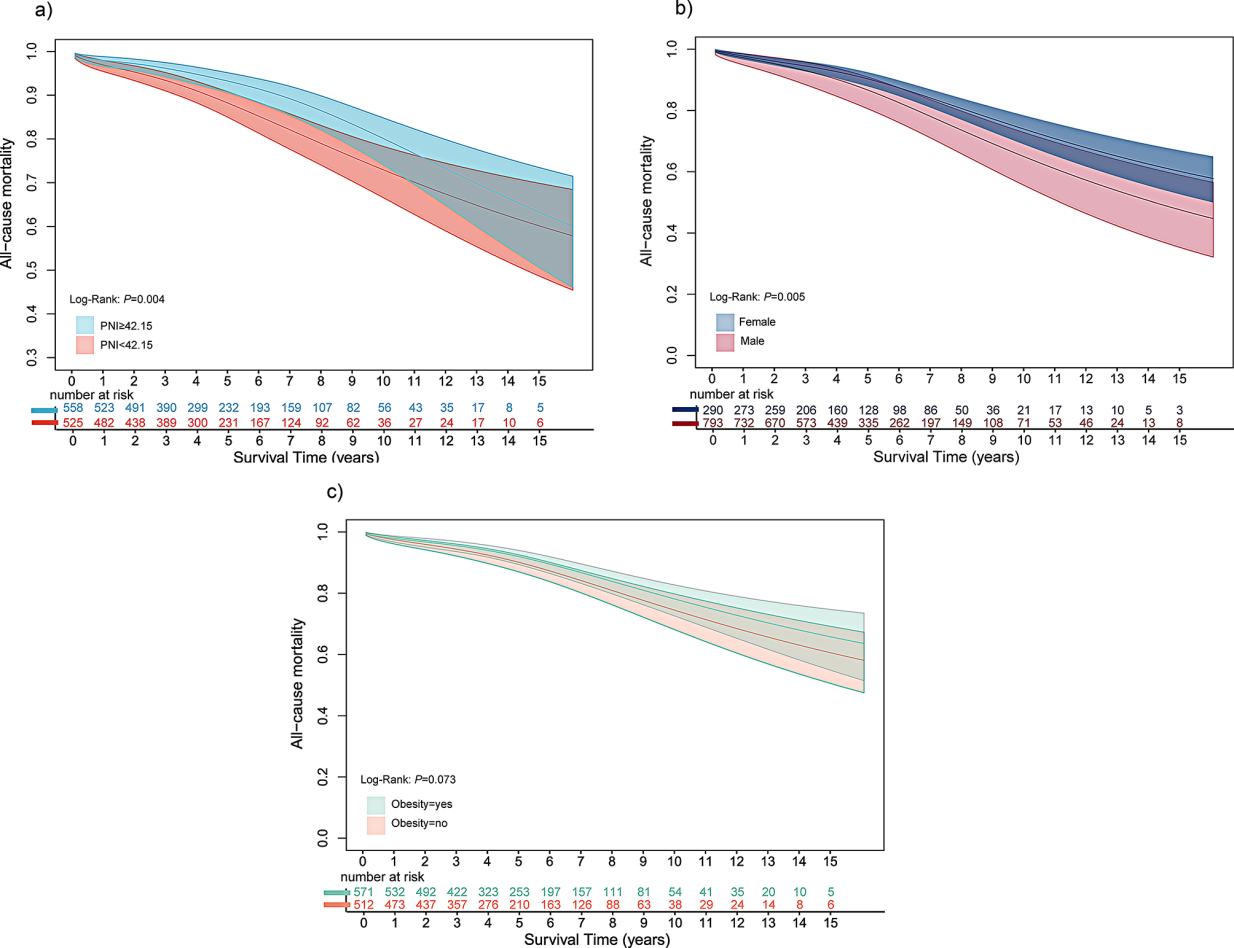
Smooth curves depicting the survival trends in older adults undergoing CABG over the years by PNI before discharge (**a**), gender (**b**), and obesity (**c**). Shaded areas represent the 95% confidence intervals (CI) for each survival curve. The number at risk, defined by Kaplan–Meier at each year, is provided for reference. PNI, prognostic nutritional index

After excluding the 90 patients who encountered short-term mortality, a cohort of 1083 patients were subjected to long-term follow-up after discharge, encompassing a duration of 225.8 months, with a median follow-up of 52.5 months. A total of 131 patients expired within this protracted follow-up period. Univariate cox regression analysis revealed that several factors were associated with an increased risk of long-term mortality, including age, gender, Ccr and PNI before discharge (Table 4). Additional multivariate cox regression analysis showed that low PNI before discharge (HR = 1.451, 95%CI: 1.012–2.082, *P* = 0.043), age, gender and Ccr were independent risk factors of long-term mortality (Fig. 3). The survival probabilities of the whole cohort at 1,3,5,10 years were 96.98%, 94.64%, 89.89%, 76.96%, respectively. Kaplan-Meier analysis revealed a significant decrease in actuarial long-term survival among patients in the lower PNI group before discharge (log-rank: *P* = 0.004) (Fig. 4a).

Further analysis was conducted on the impact of gender and obesity on long-term survival. Kaplan-Meier analysis of gender showed a higher survival rate in females than males postoperatively (log-rank: *P* = 0.005) (Fig. 4b). Interestingly, obese patients exhibited slightly higher long-term survival rates after surgery, though not significantly (log-rank: *P* = 0.073) (Fig. 4c).

**Table 4:** Univariate Cox regression analysis for long-term mortality

Variables	HR	95%CI for HR Lower-Upper	*P* value
Age	1.117	1.067–1.168	< 0.001
Gender	0.519	0.326–0.829	0.006
Ccr	0.982	0.972–0.991	< 0.001
Number of diseased coronary artery	0.730	0.525–1.015	0.062
Serum albumin before discharge	0.944	0.903–0.987	0.012
TLC before discharge	0.662	0.470–0.934	0.019
PNI before discharge	0.939	0.905–0.974	0.001
NYHA classification	1.115	0.881–1.410	0.365
Obesity status	0.889	0.766–1.033	0.124
CHD classification	0.951	0.714–1.265	0.729
Hypertension	1.001	0.688–1.457	0.996
Diabetes	1.207	0.844–1.725	0.302
Cerebrovascular disease	1.061	0.801–1.407	0.679
PVD	1.370	0.811–2.313	0.240
Valvular diseases	1.103	0.621–1.961	0.738
COPD	1.523	0.710–3.266	0.280
Preoperative AF	1.159	0.473–2.839	0.747
Pulmonary hypertension	0.954	0.640–1.422	0.816

HR, hazard ratio; CI, confidence interval; Ccr, endogenous creatinine clearance rate; TLC, total peripheral blood lymphocyte count; PNI, prognostic nutritional index; NYHA, New York heart association; CHD, coronary heart disease; PVD, peripheral vascular disease; COPD, chronic obstructive pulmonary disease; AF, atrial fibrillation

## Discussion

This study calculated and analyzed PNI at various time points and identifies low PNI in the perioperative period as an independent risk factor for both short- and long-term mortality in older adults undergoing CABG surgery. These findings suggest the potential value of PNI assessment in the prognostic evaluation of this population.

It is widely acknowledged that the mortality rate of CABG surgery tends to rise as individuals age. The findings of this study unequivocally validate this notion, and reveal an operative mortality rate of 7.7% among older adults, which is notably higher than the mortality rate of 2.52% observed in the general population in our prior research [12]. To deeper understand the factors contributing to the increased postoperative mortality rates among older adults undergoing CABG, various studies in the fields of epidemiology, pathology, nutrition and nursing have provided suggestive evidence. For instance, the prevalence of CHD is on the rise due to factors such as social progress, an aging population, and chronic metabolic diseases such as diabetes, hyperlipidemia, and hyperuricemia. Consequently, the number of CABG surgeries being conducted notably increases [13, 14]. Atherosclerosis, a direct contributor to the development of CHD, is a condition that typically manifests with advancing age. It is characterized by the presence of multiple comorbidities, a decline in organ function reserve, and diminished surgical tolerance [15, 16]. Consequently, the proportion of old individuals notably increases within the patient population. These individuals are often old and exhibit compromised cardiac, liver, and renal function reserves, rendering them more susceptible to postoperative complications [17].

Malnutrition is a common issue affecting more than 50% of hospitalized patients worldwide, particularly the old patients and chronic disease patients who are already at nutritional risk and may have experienced protein loss prior to admission [18]. This can be easily discerned by employing the PNI, which is extensively used in diverse contexts, particularly for assessing surgical risk in cancer patients [19, 20], malnourished individuals [21, 22], those with systemic inflammation [23], and those undergoing gastrointestinal surgery [24]. Nevertheless, the utilization of PNI in cardiac surgery, particularly in perioperative management of CABG surgery, remains relatively constrained. Serum albumin, a parameter for calculating PNI, plays a role in scavenging and inhibiting the activity of reactive oxygen radicals [25]. Additionally, lymphocytes, essential components of the immune system, were demonstrated as significant predictors of mortality in patients undergoing CABG [26]. The present study observed both univariate and multivariate logistic regression analyses substantiated the independent association between low PNI and postoperative short-term mortality.

A retrospective study involving 644 CHD patients undergoing CABG revealed a significant association between lower PNI levels and increased rates of both in-hospital and long-term mortality [27]. Specifically, patients with lower PNI levels had a 12-fold higher risk of in-hospital mortality (95%CI: 4.0-45.2) and a 4.9-fold higher risk of long-term (36-month) mortality (95%CI: 2.3–15.9) compared to those with higher PNI levels. Another retrospective study also reported PNI as a predictor of in-hospital postoperative mortality [26]. In accordance with their research findings, this study involving 1173 older adults further substantiated through a multivariate logistic and cox regression analysis that PNI serves as a distinct prognostic indicator for both short- and long-term mortality in old individuals. Moreover, it offers a more precise examination of the relationship between PNI and long-term all-cause mortality, with a longer follow-up period of 225.8 months. The findings underscore a significantly elevated mortality rate in the low PNI group compared to the normal PNI group.

Preoperative PNI screening can help identify malnourished older adults, leading to early targeted nutritional interventions before CABG [28]. In addition, clinicians can develop dietary plans for patients based on their PNI values before discharge and incorporate active nutritional support strategies where possible to improve patient prognosis [29]. Specifically, immunonutrient supplements, including high protein, arginine, glutamine, and ω-3 fatty acids, may be beneficial in improving the perioperative and long-term prognosis of older adults undergoing CABG with low PNI [30].

This study found that female patients had a better long-term prognosis after CABG, which contradicts much of the existing literature that suggests a worse prognosis for women. For instance, Gaudino et al.‘s study on multiple arterial graft CABG identified women as an independent risk factor for 7-year mortality [31], while Hara et al. reported higher mortality in women even at 10 years [32]. Robinson et al. (2024) pooled data from 84 studies, showing that women had a higher adjusted risk of late death and were more likely to experience major adverse cardiac events and stroke compared to men [33].

Several factors have been proposed to explain poorer outcomes in women, including smaller, more spastic coronary arteries that complicate CABG and increase the risk of graft failure. Additionally, women tend to be older and have a higher prevalence of chronic conditions, such as diabetes and hypertension [33]. However, the discrepancy in this finding, where older women showed a better long-term prognosis, could be attributed to factors like our smaller sample size, a relatively younger average age of women, a smaller difference in comorbidities between genders, and better perioperative nutritional status in women in our cohort. While this contradicts previous studies, a recent subgroup analysis of older adults with CABG found that long-term survival in women may be similar to or even better than in men [34–36], which supported our result. Furthermore, our cohort consisted of patients aged 70 and above, aligning closely with the average life expectancy in China, which is 77.3 years overall, with women living an average of 81 years and men 74.8 years as of 2019 [37]. This demographic trend may help explain the higher long-term mortality observed in men in our cohort. Further investigation is necessary, and additional studies with larger cohorts and prospective experimental designs are needed to clarify these findings and better understand gender differences in CABG outcomes.

The relationship between obesity and cardiac surgery outcomes is often referred to as the “obesity paradox” [38]. Although obesity is linked to various complications, it does not always result in worse outcomes. In this study, obese patients showed better long-term survival compared to non-obese patients, consistent with the obesity paradox. However, this paradox should be interpreted cautiously, as obesity remains associated with higher risks of cardiovascular events and mortality in certain contexts, such as severe obese and renal insufficiency [39–41].

Mariscalco et al. [42] found that obesity in cardiac surgery patients was associated with lower in-hospital mortality and fewer ischemic complications. Similarly, Romero-Corral et al. [38] and Ghanta et al. [43] suggested that mildly obese patients often have better survival outcomes, likely due to preserved lean body mass, which enhances physical and metabolic resilience. In addition, Lv Mengwei et al. [44] found an L-shaped association between BMI and the risk of death 30 days after CABG. This concept of “mild obesity”, where BMI increases primarily due to lean body mass rather than excess fat, may help explain the better outcomes observed in our obese cohort, particularly in older adults. However, severe obesity remains a risk factor for increased mortality. Notably, one study showed that despite initial survival benefits, long-term cardiovascular risks associated with obesity, such as metabolic disorders and progression of coronary artery disease, can diminish these benefits over time, often eliminating them altogether after five years [42]. In our study, with a median follow-up of 52.5 months, this suggests that the initial survival benefits for obese patients may not yet have been diminished. However, the paradox remains a critical theory and the role of obesity in older adults with CABG requires further investigation and additional studies with larger cohorts and prospective experimental designs.

### Limitations

This study necessitates the acknowledgment of certain limitations. Firstly, despite being a multicenter study, the retrospective nature of the study could not completely eliminate selective biases. The robustness of the study could be enhanced if it were designed as a prospective cohort study. Secondly, the discrepancy in hospitalization duration and data gaps resulted in variations in the timing of assessing serum albumin and total lymphocyte levels prior to discharge. This variability introduced a potential source of bias. Thirdly, patients with cirrhosis or other chronic disease, were not explicitly excluded, which may have affected serum albumin levels and, consequently, PNI values. Future research should consider excluding or separately analyzing these subgroups. Moreover, the relatively small sample size, particularly in subgroup analyses (e.g. gender and obesity), limits the generalizability of our findings. Additionally, exploratory endings are not analysed in sufficient depth, and more in-depth analyses are needed. Larger studies with diverse populations are necessary. In addition, operative factors such as prolonged CPB times and surgical stress can significantly lower serum albumin levels, potentially biasing PNI before discharge and the association with long-term mortality. Future studies with dynamic monitoring of PNI and postoperative albumin recovery are needed to better understand these relationships and minimize potential bias. Lastly, the lack of dynamic data on PNI during the follow-up period introduced certain bias in the long-term outcome analysis. To provide a more comprehensive understanding, future studies could incorporate real-time monitoring of PNI changes over an extended follow-up duration.

## Conclusions

Firstly, this study underscores that older adults exhibit a higher mortality rate following CABG surgery. Secondly, it establishes low PNI as a significant risk factor for both short- and long-term mortality in older adults undergoing CABG surgery. Future research could focus on conducting prospective cohort studies with larger cohorts to further validate the relationship between PNI and mortality in older adults receiving CABG and whether targeted nutritional intervention strategies based on PNI are beneficial.

## Data Availability

The datasets used and/or analyzed in the current study are available from the corresponding authors upon reasonable request.

## Abbreviations

AF: Atrial fibrillation
BMI: Body mass index
BSA: Body surface area
CABG: Coronary artery bypass grafting
Ccr: Creatinine clearance rate
CHD: Coronary heart disease
CI: Confidence interval
COPD: Chronic obstructive pulmonary disease
CPB: Cardiopulmonary bypass
HR: Hazard ratio
IABP: Intra-aortic balloon pump
IQR: Interquartile range
LVEF: Left ventricular ejection fraction
NYHA: New York heart association
OR: Odds ratio
PCI: Percutaneous coronary intervention
PNI: Prognostic nutritional index
PVD: Peripheral vascular disease
ROC: Receiver’s operating characteristic
SD: Standard deviation
TLC: Total peripheral blood lymphocyte count
